# Crystal structure of a chloride-bridged copper(II) dimer: piperazine-1,4-dium bis­(di-μ-chlorido-bis[(4-carboxypyridine-2-carboxyl­ato-κ^2^ *N*,*O* ^2^)chlorido­cuprate(II)]

**DOI:** 10.1107/S2056989017001013

**Published:** 2017-01-27

**Authors:** Bassey Enyi Inah, Ayi Anyama Ayi, Amit Adhikary

**Affiliations:** a Inorganic Materials Chemistry Laboratory, Department of Pure and Applied Chemistry, University of Calabar, P.M.B. 1115-Calabar, Nigeria; b Department of Chemistry, Missouri University of Science and Technology, Rolla, MO 65409, USA

**Keywords:** crystal structure, solvothermal synthesis, coordination polymer, centrosymmetric dimer

## Abstract

Crystals of a new dimeric chloride-bridged cuprate(II) derived from pyridine-2,4-di­carb­oxy­lic acid were obtained solvothermally in the presence of piperazine and hydro­chloric acid. The crystal structure determination of the title salt, (C_4_H_12_N_2_)[Cu_2_(C_7_H_4_NO_4_)_2_Cl_4_], revealed one of the carboxyl groups of the original pyridine-2,4-di­carb­oxy­lic acid ligand to be protonated, whereas the other is deprotonated and binds together with the pyridine N atom to the Cu^II^ atom. The coordination environment of the Cu^II^ atom is distorted square-pyramidal. One of the chloride ligands bridges two metal cations to form a centrosymmetric dimer with two different Cu—Cl distances of 2.2632 (8) and 2.7853 (8) Å, whereby the longer distance is associated with the apical ligand. The remaining chloride ligand is terminal at one of the basal positions, with a distance of 2.2272 (9) Å. In the crystal, the dimers are linked by inter­molecular O—H⋯O hydrogen bonds, together with N—H⋯O and N—H⋯Cl inter­actions involving the centrosymmetric organic cation, into a three-dimensional supra­molecular network. Further but weaker C—H⋯O and C—H⋯Cl inter­actions consolidate the packing.

## Abstract 2

A new centrosymmetric chloride-bridged Cu^II^ dimeric anion, in which one carboxyl group of the pyridine-2,4-di­carb­oxy­lic acid ligand remains protonated, is present in the title structure, together with a diprotonated piperazine as a charge-compensating agent.

## Chemical context   

In recent times, research on coordination polymers, popularly known as metal–organic frameworks (MOFs), have received great attention, not only for their potential applications in the area of gas storage, ion-exchange, non-linear optics, mol­ecular sieves, catalysis, magnetism, and mol­ecular sensing (Yaghi *et al.*, 2003 ▸; Ockwig *et al.*, 2005 ▸; Wang *et al.*, 2005 ▸; Carlucci *et al.*, 2003 ▸; Hill *et al.*, 2005 ▸), but also for their rich structural chemistry (Li *et al.*, 2016 ▸; Eddaoudi *et al.*, 2015 ▸). In the design of compounds with metal–organic frameworks, versatile carboxyl­ate ligands, derived from 1,4-benzene­dicarb­oxy­lic acid, 1,3,5-benzene­tri­carb­oxy­lic acid, 1,2,4,5-benzene­tetra­carb­oxy­lic acid or pyridine-2,4-di­carb­oxy­lic acid, have frequently been used owing to their abundant carboxyl­ate groups possessing high affinity to metal cations (Li *et al.*, 2004 ▸; Shi *et al.*, 2004 ▸; Gutschke *et al.*, 2001 ▸; Tao *et al.*, 2000 ▸). A number of novel metal–organic frameworks have been constructed using di- or multi­carboxyl­ate ligands as linkers. Most of the reported MOF materials have been synthesized using solvothermal or hydro­thermal synthetic conditions, often by using sealed autoclaves. These techniques have also been found to play an important role in preparing robust and stable inorganic compounds with open frameworks (Rao *et al.*, 2001 ▸; Eddaoudi *et al.*, 2001 ▸). The fact that the solubility of the reactants increases under hydro­thermal methods makes the reaction more likely to occur at lower temperatures, with the formation of polymeric units through mol­ecular building blocks (Zhao *et al.*, 2007 ▸). Small changes in one or more of the reaction variables, such as temperature, time, pH or the solvent type, can have a profound influence on the product. In some cases, organic amines or alkyl­ammonium cations are used as templates and/or structure-directing agents in the crystallization process of framework solids (Jiang *et al.*, 1998 ▸; Cheetham *et al.*, 1999 ▸). In the course of our investigations, we were inter­ested in using pyridine-2,4-di­carb­oxy­lic acid as a source of *N-* and *O-*donors, in synthesizing a coordination polymer in an acidic medium under solvothermal conditions and in the presence of piperazine as an organic amine. In this context we report on the synthesis and crystal structure of the title compound (C_4_H_12_N_2_)[Cu_2_(C_7_H_4_NO_4_)_2_Cl_4_], (I).
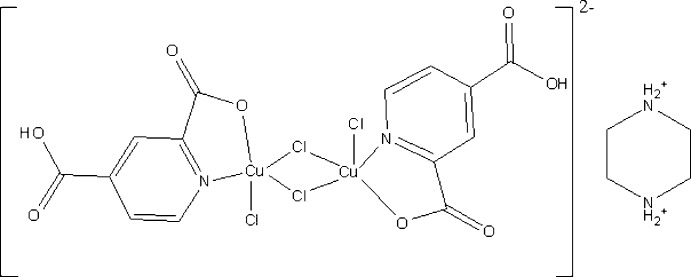


## Structural commentary   

**Figure 1:**
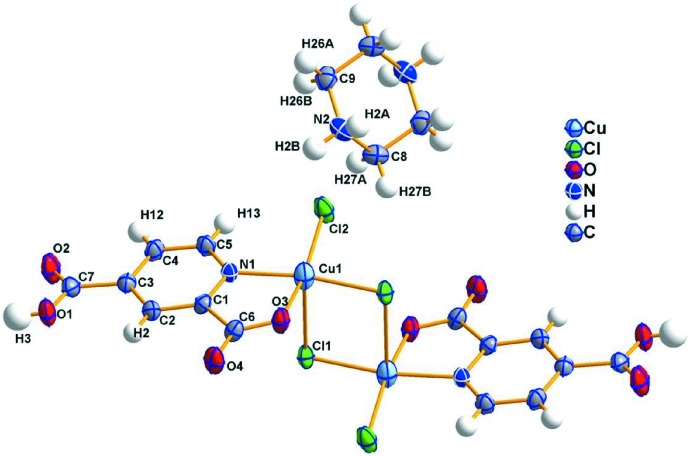
The mol­ecular structures of the cationic and anionic components of (I). Displacement ellipsoids are drawn at the 50% probability level. The non-labelled atoms are related to the labelled atoms by −*x* + 2, −*y* + 2, −*z* + 1;.

The mol­ecular structure of (I) showing the numbering scheme is presented in Fig. 1 ▸. The copper(II) atom is chelated by the O atom (O3) of the deprotonated carb­oxy­lic group and the pyridine N atom (N1) of the organic ligand, forming a five-membered chelate ring Cu1–N1–C1–C6–O3. Two bridging and one terminal chlorido ligands complete the distorted square-pyramidal coordination of the metal cation. The arrangement of the chlorido ligands is such that Cl1 is doubly bridging the two metal cations into a centrosymmetric dimer through edge-sharing. The apical Cu—Cl1(−*x* + 2, −*y* + 2, −*z* + 1) bond length of 2.7853 (9) Å is significantly longer than the other bridging Cu—Cl bond with a length of 2.2632 (8) Å. The square plane is formed by N1 and O3, both from the pyridine-2,4-di­carboxyl­ate anion, Cl1 from the bridging chlorido ligand and Cl2 of the terminal chlorido ligand [2.2272 (9) Å]. This type of coordination has been previously described as a transition state between 4- and 5-coordinate (Qi *et al.*, 2009 ▸). The distortion index (*τ*) assuming a square-pyramidal environment was calculated as 0.08 using the formula, *τ* = (*β* − *α*)/60 (*α*, *β* are the largest valence angles) proposed by Addison *et al.* (1984 ▸), which indicates only slight distortions from the ideal value where *τ* = 0. The Cu⋯Cu distance in the dimer is 3.5946 (9) Å, with an Cu—Cl—Cu bond angle of 90.19 (3)° and a Cl⋯Cl separation of 3.5831 (14) Å. The Cu—N and Cu—O bond lengths are 2.013 (2) and 1.963 (2) Å, respectively, and are in good agreement with similar compounds reported in the literature (Goddard *et al.*, 1990 ▸; Tynan *et al.*, 2005 ▸; Han *et al.*, 2008 ▸; Liu *et al.*, 2009 ▸; Qi *et al.*, 2009 ▸). The chelate angle O3—Cu—N1 of 81.34 (9)° is, as expected, smaller than the N1—Cu—Cl1 and O3—Cu—Cl2 bond angles of 170.22 (7) and 165.23 (8)°, respectively. The inorganic anion has a charge of −2 that is compensated by the incorporation of a fully protonated piperazine mol­ecule in the structure. The latter is located about an inversion centre.

## Supra­molecular features   

**Figure 2:**
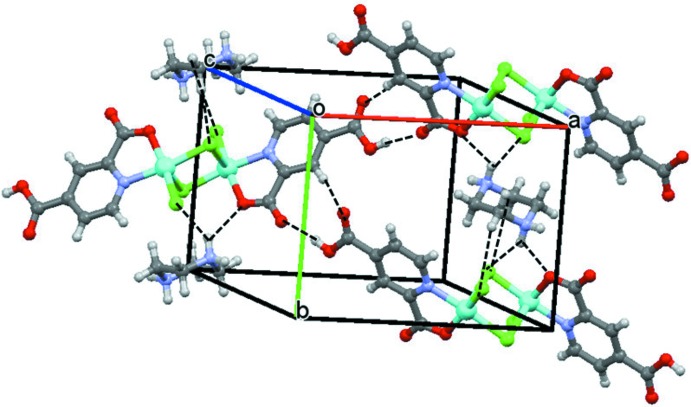
The crystal structure of (I), showing O—H⋯O, N—H⋯O and N—H⋯Cl hydrogen-bonding inter­actions as dashed lines (see Table 1 ▸ for numerical details).

The centrosymmetric dimers are linked by pairs of (carbox­yl)O1—H3⋯O4(carboxyl­ate) hydrogen bonds to form sheets parallel (100). The protonated centrosymmetric amine cations are situated between the sheets and are connected through N2—H⋯O2 inter­actions to one of the carbonyl oxygen atoms and various N—H⋯Cl inter­actions into a three-dimensional network (Table 1 ▸, Fig. 2 ▸). The carbonyl oxygen atom O2 also acts as a hydrogen-bond acceptor from pyridyl C—H groups (C2—H2⋯O2 and C4—H12⋯O2). These inter­actions, together with C—H⋯Cl inter­actions, further stabilize the three-dimensional supra­molecular network structure.

## Database survey   

There are several copper(II) dimeric compounds in which the copper atoms are bridged by chlorido ligands (Marsh *et al.*, 1983 ▸; Puschmann *et al.*, 2001 ▸; Li *et al.*, 2006 ▸; Lee, *et al.*, 2008 ▸; Han *et al.*, 2008 ▸; Øien *et al.*, 2013 ▸; Choubey *et al.*, 2015 ▸; Golchoubian & Nateghi 2016 ▸; Liu *et al.*, 2009 ▸). A search of the Cambridge Structural Database (Version 5.38, November 2016; Groom *et al.*, 2016 ▸), revealed numerous di-μ-chlorido bridged copper(II) compounds constructed with ligands having -*N,O*- donor atoms (Kapoor *et al.*, 2002 ▸, 2004 ▸; Damous *et al.*, 2013 ▸; Lumb *et al.*, 2013 ▸; Smolentsev *et al.*, 2014 ▸; Qureshi *et al.*, 2016 ▸). However, the search did not reveal related complexes derived from pyridine-2,4-di­carb­oxy­lic acid and piperazine.

## Synthesis and crystallization   

The syntheses were carried out in Ace pressure tubes (15 cm^3^) and heated in programmable ovens. The reagents used for syntheses were obtained from Aldrich (Analar grade) and used without further purification. In a typical synthesis of (I), Cu(CH_3_COO)_2_·2H_2_O (0.1996 g, 1.0 mmol) was stirred together with pyridine-2,4-di­carb­oxy­lic acid (0.1671 g, 1.0 mmol) in 3.3 cm^3^ of *n*-butanol. This was followed by the addition of piperazine (0.940 g, 1.0 mmol) and the pH of the solution was adjusted to 2 by dropwise addition of 0.16 cm^3^ of conc. HCl. The resultant mixture was homogenized for 15 min before transferring into the reaction vessel and heated in an oven at 393 K for 48 h. The product, a crop of bluish crystalline material, was washed with distilled water and air-dried.

## Refinement   

Crystal data, data collection and structure refinement details are summarized in Table 2 ▸. C-bound H atoms were treated as riding atoms, with C—H distances of 0.93 Å (aromatic) and 0.97 Å (aliphatic), and with *U*_iso_(H) = 1.2*U*_eq_(C). N- and O-bound H atoms were located in difference maps and were refined with N—H distances of 0.89 Å and O—H distances of 0.82 Å, and with *U*_iso_(H) = 1.2*U*_eq_(N) and *U*_iso_(H) = 1.5*U*_eq_(O), respectively.

## Supplementary Material

Crystal structure: contains datablock(s) I. DOI: 10.1107/S2056989017001013/wm5354sup1.cif

Structure factors: contains datablock(s) I. DOI: 10.1107/S2056989017001013/wm5354Isup2.hkl

CCDC reference: 1497751

Additional supporting information:  crystallographic information; 3D view; checkCIF report

## Figures and Tables

**Table 1:** Hydrogen-bond geometry (Å, °)

*D*—H⋯*A*	*D*—H	H⋯*A*	*D*⋯*A*	*D*—H⋯*A*
O1—H3⋯O4^i^	0.82	1.79	2.603 (3)	171
N2—H2*A*⋯Cl1^ii^	0.89	2.78	3.562 (3)	147
N2—H2*A*⋯O3^ii^	0.89	2.22	2.861 (3)	129
N2—H2*B*⋯Cl1^iii^	0.89	2.69	3.414 (3)	139
N2—H2*B*⋯Cl2^iii^	0.89	2.69	3.360 (3)	133
C2—H2⋯O2^iv^	0.93	2.49	3.402 (4)	169
C4—H12⋯O2^v^	0.93	2.56	3.362 (4)	145
C5—H13⋯Cl2	0.93	2.71	3.269 (3)	119
C8—H27*A*⋯Cl1^vi^	0.97	2.72	3.561 (3)	146
C8—H27*B*⋯Cl2^vii^	0.97	2.81	3.599 (3)	139
C9—H26*A*⋯O2^viii^	0.97	2.56	3.509 (4)	165
C9—H26*B*⋯Cl1^vi^	0.97	2.93	3.713 (3)	139
C9—H26*B*⋯Cl2^iii^	0.97	2.93	3.491 (4)	118

Symmetry codes: (i) 
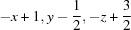
; (ii) 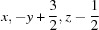; (iii) 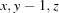; (iv) 
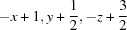
; (v) 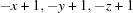; (vi) 
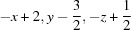
; (vii) 
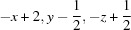
; (viii) 
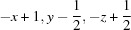
.

**Table 2:** Experimental details

Crystal data	
Chemical formula	(C_4_H_12_N_2_)[Cu_2_(C_7_H_4_NO_4_)_2_Cl_4_]
*M*_r_	689.26
Crystal system, space group	Monoclinic, *P*2_1_/*c*
Temperature (K)	298
*a*, *b*, *c* (Å)	11.639 (3), 9.224 (2), 11.423 (3)
β (°)	105.211 (3)
*V* (Å^3^)	1183.4 (5)
*Z*	2
Radiation type	Mo *K*α
μ (mm^−1^)	2.30
Crystal size (mm)	0.05 × 0.02 × 0.02

Data collection	
Diffractometer	Bruker *SMART* *APEX* CCD area detector
Absorption correction	Multi-scan (*SADABS*; Bruker, 2008 ▸)
*T*_min_, *T*_max_	0.946, 0.955
No. of measured, independent and observed [*I* > 2σ(*I*)] reflections	14235, 2923, 2392
*R*_int_	0.050
(sin θ/λ)_max_ (Å^−1^)	0.666

Refinement	
*R*[*F* ^2^ > 2σ(*F* ^2^)], *wR*(*F* ^2^), *S*	0.039, 0.127, 0.86
No. of reflections	2923
No. of parameters	164
H-atom treatment	H-atom parameters constrained
Δρ_max_, Δρ_min_ (e Å^−3^)	0.55, −0.31

Computer programs: *SMART* and *SAINT* (Bruker, 2008 ▸), *SHELXS97* (Sheldrick, 2008 ▸), *SHELXL2014* (Sheldrick, 2015 ▸), *SHELXTL* (Sheldrick, 2008 ▸) and *Mercury* (Macrae *et al.*, 2006 ▸).

## supplementary crystallographic information

### Crystal data

**Table:**

(C_4_H_12_N_2_)[Cu_2_(C_7_H_4_NO_4_)_2_Cl_4_]	*F*(000) = 692
*M**_r_* = 689.26	*D*_x_ = 1.934 Mg m^−^^3^
Monoclinic, *P*2_1_/*c*	Mo *K*α radiation, λ = 0.71073 Å
*a* = 11.639 (3) Å	Cell parameters from 1016 reflections
*b* = 9.224 (2) Å	θ = 2.9–26.8°
*c* = 11.423 (3) Å	µ = 2.30 mm^−^^1^
β = 105.211 (3)°	*T* = 298 K
*V* = 1183.4 (5) Å^3^	Rod, blue
*Z* = 2	0.05 × 0.02 × 0.02 mm

### Data collection

**Table:**

Bruker SMART APEX CCD area detector diffractometer	2392 reflections with *I* > 2σ(*I*)
ω scans	*R*_int_ = 0.050
Absorption correction: multi-scan (*SADABS*; Bruker, 2008)	θ_max_ = 28.3°, θ_min_ = 2.9°
*T*_min_ = 0.946, *T*_max_ = 0.955	*h* = −15→15
14235 measured reflections	*k* = −12→12
2923 independent reflections	*l* = −15→15

### Refinement

**Table:**

Refinement on *F*^2^	0 restraints
Least-squares matrix: full	Hydrogen site location: inferred from neighbouring sites
*R*[*F*^2^ > 2σ(*F*^2^)] = 0.039	H-atom parameters constrained
*wR*(*F*^2^) = 0.127	*w* = 1/[σ^2^(*F*_o_^2^) + (0.1*P*)^2^] where *P* = (*F*_o_^2^ + 2*F*_c_^2^)/3
*S* = 0.86	(Δ/σ)_max_ = 0.001
2923 reflections	Δρ_max_ = 0.55 e Å^−^^3^
164 parameters	Δρ_min_ = −0.31 e Å^−^^3^

### Special details

**Table:**

Geometry. All esds (except the esd in the dihedral angle between two l.s. planes) are estimated using the full covariance matrix. The cell esds are taken into account individually in the estimation of esds in distances, angles and torsion angles; correlations between esds in cell parameters are only used when they are defined by crystal symmetry. An approximate (isotropic) treatment of cell esds is used for estimating esds involving l.s. planes.

### Fractional atomic coordinates and isotropic or equivalent isotropic displacement parameters (Å^2^)

**Table:**

	*x*	*y*	*z*	*U*_iso_*/*U*_eq_	
Cu1	0.84456 (3)	1.01123 (4)	0.42216 (3)	0.02632 (15)	
Cl1	0.99944 (6)	1.14015 (7)	0.39127 (7)	0.02963 (19)	
Cl2	0.82884 (8)	0.86943 (9)	0.26090 (7)	0.0431 (2)	
O3	0.82154 (19)	1.1592 (2)	0.5374 (2)	0.0326 (5)	
O4	0.7301 (2)	1.2063 (2)	0.6797 (2)	0.0411 (6)	
N1	0.7122 (2)	0.9149 (2)	0.4767 (2)	0.0244 (5)	
O1	0.4230 (2)	0.8203 (3)	0.7170 (2)	0.0412 (6)	
H3	0.379334	0.776566	0.750692	0.062*	
C6	0.7489 (2)	1.1293 (3)	0.5993 (3)	0.0259 (6)	
N2	0.9556 (2)	0.0777 (3)	0.0881 (2)	0.0358 (6)	
H2A	0.959588	0.169430	0.065288	0.043*	
H2B	0.926778	0.077335	0.152968	0.043*	
C1	0.6847 (3)	0.9859 (3)	0.5683 (3)	0.0246 (6)	
O2	0.4234 (2)	0.6091 (3)	0.6208 (2)	0.0468 (6)	
C2	0.6029 (2)	0.9326 (3)	0.6261 (3)	0.0261 (6)	
H2	0.584506	0.983758	0.688974	0.031*	
C8	1.0765 (3)	0.0148 (3)	0.1210 (3)	0.0323 (7)	
H27A	1.073784	−0.081047	0.155093	0.039*	
H27B	1.128410	0.074758	0.182456	0.039*	
C7	0.4583 (3)	0.7319 (3)	0.6429 (3)	0.0310 (6)	
C4	0.5776 (3)	0.7273 (3)	0.4928 (3)	0.0308 (6)	
H12	0.542510	0.638592	0.465717	0.037*	
C5	0.6597 (3)	0.7892 (3)	0.4394 (3)	0.0294 (6)	
H13	0.678695	0.741211	0.375346	0.035*	
C3	0.5487 (3)	0.7998 (3)	0.5872 (3)	0.0268 (6)	
C9	0.8733 (3)	−0.0041 (3)	−0.0119 (3)	0.0367 (7)	
H26A	0.796776	0.044487	−0.034859	0.044*	
H26B	0.861083	−0.100761	0.016075	0.044*	

### Atomic displacement parameters (Å^2^)

**Table:**

	*U*^11^	*U*^22^	*U*^33^	*U*^12^	*U*^13^	*U*^23^
Cu1	0.0282 (2)	0.0222 (2)	0.0335 (2)	−0.00475 (12)	0.01701 (17)	−0.00548 (13)
Cl1	0.0306 (4)	0.0252 (3)	0.0368 (4)	−0.0053 (3)	0.0155 (3)	0.0021 (3)
Cl2	0.0595 (5)	0.0385 (4)	0.0410 (5)	−0.0166 (4)	0.0305 (4)	−0.0152 (3)
O3	0.0350 (11)	0.0255 (10)	0.0437 (12)	−0.0075 (9)	0.0215 (10)	−0.0092 (9)
O4	0.0424 (13)	0.0372 (12)	0.0520 (14)	−0.0066 (10)	0.0272 (11)	−0.0194 (10)
N1	0.0249 (11)	0.0239 (12)	0.0270 (11)	−0.0007 (9)	0.0112 (9)	−0.0022 (9)
O1	0.0441 (14)	0.0401 (13)	0.0497 (14)	−0.0112 (11)	0.0306 (12)	−0.0032 (11)
C6	0.0219 (13)	0.0217 (13)	0.0348 (15)	−0.0001 (10)	0.0086 (11)	−0.0051 (11)
N2	0.0454 (15)	0.0311 (14)	0.0334 (13)	0.0120 (12)	0.0150 (12)	0.0001 (11)
C1	0.0209 (12)	0.0247 (14)	0.0281 (14)	0.0024 (10)	0.0064 (11)	−0.0005 (10)
O2	0.0573 (16)	0.0355 (12)	0.0581 (16)	−0.0167 (11)	0.0337 (13)	−0.0040 (11)
C2	0.0238 (13)	0.0291 (14)	0.0264 (13)	−0.0003 (11)	0.0085 (11)	−0.0007 (11)
C8	0.0422 (18)	0.0228 (14)	0.0280 (15)	0.0016 (12)	0.0027 (14)	−0.0002 (11)
C7	0.0290 (14)	0.0344 (16)	0.0291 (14)	−0.0046 (12)	0.0068 (12)	0.0058 (12)
C4	0.0309 (15)	0.0276 (14)	0.0350 (15)	−0.0075 (12)	0.0106 (13)	−0.0022 (12)
C5	0.0320 (15)	0.0288 (15)	0.0300 (14)	−0.0037 (12)	0.0130 (12)	−0.0053 (11)
C3	0.0240 (13)	0.0276 (14)	0.0291 (14)	0.0002 (11)	0.0076 (11)	0.0037 (11)
C9	0.0313 (17)	0.0374 (18)	0.0416 (19)	0.0024 (12)	0.0099 (15)	0.0037 (13)

### Geometric parameters (Å, º)

**Table:**

Cu1—O3	1.963 (2)	N2—H2B	0.8900
Cu1—N1	2.013 (2)	C1—C2	1.384 (4)
Cu1—Cl2	2.2272 (9)	O2—C7	1.207 (4)
Cu1—Cl1	2.2632 (8)	C2—C3	1.396 (4)
Cu1—Cl1^i^	2.7853 (9)	C2—H2	0.9300
O3—C6	1.267 (3)	C8—C9^ii^	1.512 (5)
O4—C6	1.225 (3)	C8—H27A	0.9700
N1—C5	1.327 (4)	C8—H27B	0.9700
N1—C1	1.343 (4)	C7—C3	1.502 (4)
O1—C7	1.316 (4)	C4—C3	1.383 (4)
O1—H3	0.8200	C4—C5	1.385 (4)
C6—C1	1.515 (4)	C4—H12	0.9300
N2—C8	1.476 (4)	C5—H13	0.9300
N2—C9	1.490 (4)	C9—H26A	0.9700
N2—H2A	0.8900	C9—H26B	0.9700
			
O3—Cu1—N1	81.34 (9)	C3—C2—H2	121.0
O3—Cu1—Cl2	165.23 (8)	N2—C8—C9^ii^	111.4 (3)
N1—Cu1—Cl2	95.35 (7)	N2—C8—H27A	109.4
O3—Cu1—Cl1	89.63 (6)	C9^ii^—C8—H27A	109.4
N1—Cu1—Cl1	170.22 (7)	N2—C8—H27B	109.4
Cl2—Cu1—Cl1	94.33 (3)	C9^ii^—C8—H27B	109.4
C6—O3—Cu1	116.83 (18)	H27A—C8—H27B	108.0
C5—N1—C1	119.5 (2)	O2—C7—O1	124.8 (3)
C5—N1—Cu1	127.7 (2)	O2—C7—C3	122.5 (3)
C1—N1—Cu1	112.59 (18)	O1—C7—C3	112.7 (3)
C7—O1—H3	109.5	C3—C4—C5	118.7 (3)
O4—C6—O3	124.7 (3)	C3—C4—H12	120.6
O4—C6—C1	120.5 (3)	C5—C4—H12	120.6
O3—C6—C1	114.8 (2)	N1—C5—C4	122.1 (3)
C8—N2—C9	112.0 (2)	N1—C5—H13	118.9
C8—N2—H2A	109.2	C4—C5—H13	118.9
C9—N2—H2A	109.2	C4—C3—C2	119.4 (3)
C8—N2—H2B	109.2	C4—C3—C7	118.0 (3)
C9—N2—H2B	109.2	C2—C3—C7	122.6 (3)
H2A—N2—H2B	107.9	N2—C9—C8^ii^	110.8 (3)
N1—C1—C2	122.2 (3)	N2—C9—H26A	109.5
N1—C1—C6	113.9 (3)	C8^ii^—C9—H26A	109.5
C2—C1—C6	123.9 (3)	N2—C9—H26B	109.5
C1—C2—C3	118.0 (3)	C8^ii^—C9—H26B	109.5
C1—C2—H2	121.0	H26A—C9—H26B	108.1

Symmetry codes: (i) −*x*+2, −*y*+2, −*z*+1; (ii) −*x*+2, −*y*, −*z*.

### Hydrogen-bond geometry (Å, º)

**Table:**

*D*—H···*A*	*D*—H	H···*A*	*D*···*A*	*D*—H···*A*
O1—H3···O4^iii^	0.82	1.79	2.603 (3)	171
N2—H2*A*···Cl1^iv^	0.89	2.78	3.562 (3)	147
N2—H2*A*···O3^iv^	0.89	2.22	2.861 (3)	129
N2—H2*B*···Cl1^v^	0.89	2.69	3.414 (3)	139
N2—H2*B*···Cl2^v^	0.89	2.69	3.360 (3)	133
C2—H2···O2^vi^	0.93	2.49	3.402 (4)	169
C4—H12···O2^vii^	0.93	2.56	3.362 (4)	145
C5—H13···Cl2	0.93	2.71	3.269 (3)	119
C8—H27*A*···Cl1^viii^	0.97	2.72	3.561 (3)	146
C8—H27*B*···Cl2^ix^	0.97	2.81	3.599 (3)	139
C9—H26*A*···O2^x^	0.97	2.56	3.509 (4)	165
C9—H26*B*···Cl1^viii^	0.97	2.93	3.713 (3)	139
C9—H26*B*···Cl2^v^	0.97	2.93	3.491 (4)	118

Symmetry codes: (iii) −*x*+1, *y*−1/2, −*z*+3/2; (iv) *x*, −*y*+3/2, *z*−1/2; (v) *x*, *y*−1, *z*; (vi) −*x*+1, *y*+1/2, −*z*+3/2; (vii) −*x*+1, −*y*+1, −*z*+1; (viii) −*x*+2, *y*−3/2, −*z*+1/2; (ix) −*x*+2, *y*−1/2, −*z*+1/2; (x) −*x*+1, *y*−1/2, −*z*+1/2.
